# C4d as a Screening Tool and an Independent Predictor of Clinical Outcomes in Lupus Nephritis and IgA Nephropathy

**DOI:** 10.3389/fmed.2022.832998

**Published:** 2022-01-31

**Authors:** Xiaoqian Yang, Yanhong Yuan, Xinghua Shao, Huihua Pang, Xiajing Che, Liou Cao, Minfang Zhang, Yao Xu, Zhaohui Ni, Chaojun Qi, Qin Wang, Shan Mou

**Affiliations:** Department of Nephrology, Renji Hospital, School of Medicine, Shanghai Jiao Tong University, Shanghai, China

**Keywords:** complement, C4d, lupus nephritis, IgA nephropathy, relapse, disease progression

## Abstract

**Background:**

As an indispensable marker of complement cascades activation, C4d was confirmed of its crucial role in the pathogenesis of both lupus nephritis (LN) and IgA nephropathy (IgAN). While the studies directly comparing the diagnostic value, and outcomes predicting function of C4d between LN and IgAN are still absent.

**Methods:**

A cohort of 120 LN patients, 120 IgAN patients who were diagnosed by renal biopsy between January 2015 and December 2017 and 24 healthy age matched controls were prospectively analyzed. The patients were followed till December 2020. The outcomes were adverse disease treatment response (disease relapse) and kidney disease progression event (decline of estimated glomerular filtration rate by more than 20% or end-stage kidney disease). The renal C4d deposition proportion and pattern were compared between IgAN and LN patients. In addition, the relationship between renal C4d deposition and disease subtypes, disease relapse as well as disease progression for LN and IgAN patients were also analyzed.

**Results:**

The LN, IgAN patients and healthy controls were well matched in ages. The follow-up period was 38.5 (30.3–60.8) months for LN patients and 45.0 (30.5–57.0) months for IgAN patients. 78 patients (65.0%) with LN had renal C4d deposition, compared with only 39 IgAN patients (32.5%) with C4d deposition in renal tissues (*P* < 0.001). The LN patients shared different renal C4d distribution patterns with IgAN patients. Compared with IgAN patients, the C4d deposition in LN patients was significantly more in renal glomerulus (*P* < 0.001) and less in renal tubules (*P* = 0.003). For disease subtypes, renal C4d deposition was especially strong in class V membranous LN and IgAN with tubulointerstitial fibrosis (T1/T2) lesions. Renal C4d deposition was independently correlated with the disease relapse of LN patients (HR = 1.007, *P* = 0.040), and acted as an independent predictor of disease progression during the follow-up period for IgAN patients (HR = 1.821, *P* = 0.040).

**Conclusions:**

Renal C4d distribution proportion and pattern differed between LN and IgAN patients. The presence of C4d in renal tissue acted as an independent predictor of relapse for LN patients and disease progression for IgAN patients.

## Introduction

Systemic lupus erythematosus (SLE) is an autoimmune disease, and up to 60% of SLE patients developed Lupus nephritis (LN) during the course of the disease (1). Immune complex deposition and complement activation in the kidney significantly contributed to the pathogenesis of LN (2–4). IgA nephropathy (IgAN) is the most common primary glomerulonephritis worldwide, and the diagnostic histologic hallmark for IgAN is dominant or codominant IgA staining on kidney biopsy (5). Complement activation through alternative and lectin pathways might play significant roles in the pathogenesis of IgAN via upregulating local inflammatory responses and glomerular injury (6, 7).

Although both LN and IgAN are immune complex-mediated diseases, the pathogenesis, their etiologies, and the therapeutic strategies are different. However, co-occurrences of SLE and IgAN were found in some patients (8–10). Shared genetics between immune-related diseases may account for this in some extent (11). In this case, it is significant to find a reliable screening and diagnostic tool to differentiate the renal disease in SLE patients between LN and IgAN, which has important implications on therapeutic regimens to be adopted, and further influences the disease prognosis in both short and long term.

Dysfunction of the complement system plays a crucial role in the development of LN and IgAN (6, 12). As the most polymorphic protein in complement system, C4d is considered as an indispensable marker of complement cascades activation (13). Meanwhile, C4d has a much longer half-life, and appears to persist for weeks in the tissue due to covalent binding to the tissue structures (14), indicating its great potential for the clinical application as a marker for the progression of LN and IgAN. Previous studies indicated that renal C4d deposition in LN is associated with the LN subtype, the immune complex deposits, and the glomerular microthrombosis (15, 16). For IgAN, C4d deposition in renal tissue has been associated with aggressive histology and poor renal outcome (17, 18). However, studies specific for comparing the roles of C4d in LN and IgAN are still absent. Based on the limitations of the existing studies, the aim of our study is to compare the screening and diagnostic value, meanwhile, the clinical and prognostic implications of renal C4d deposition between LN and IgAN patients.

## Materials and Methods

### Study Population

Patients who were diagnosed of LN or IgAN by renal biopsy between January 2015 and December 2017 from the Department of Nephrology, Ren Ji Hospital, Shanghai, China were included in the study. The exclusion criteria were as follows: (1) age less than 18 years; (2) LN or IgAN of transplant kidney; (3) co-occurrence of severe systemic disease that may affect the prognosis (such as heart failure, malignant tumor); (4) lost to follow-up. The patients had regular follow-up visits till December 2020 at intervals of at least 3–6 months. And the age matched controls were healthy blood donors without indicators of renal disease. Finally a total of 264 subjects were enrolled, including 120 LN patients, 120 IgAN patients and 24 healthy controls. All the data were collected prospectively.

### Outcomes

The analysis included disease treatment and disease progression outcomes. Adverse disease treatment outcome was disease relapse at the sixth month after the initiation of therapy. The disease progression event was defined as a decline of eGFR by more than 20% or end-stage kidney disease during the follow-up period. End-stage kidney disease was defined as eGFR <15 mL/min/1.73 m^2^ or initiation of renal replacement therapy.

### Definitions

The treatment response was evaluated at the sixth month after the initiation of therapy for LN and IgAN patients. In the LN patients, the response criteria were defined according to the KIDGO guideline criteria (19). Complete response (CR): Return of serum creatinine (Scr) to previous baseline, plus a decline in the urine protein-creatinine ratio (uPCR) to <500 mg/g (<50 mg/mmol). Partial response (PR): Stabilization (±25%), or improvement of Scr, but not to normal, plus a <50% decrease in uPCR. If there was nephrotic-range proteinuria (uPCR ≥3,000 mg/g [≥300 mg/mmol]), improvement requires a ≥50% reduction in uPCR, and a uPCR <3,000 mg/g [<300 mg/mmol]. Non-response (NR): Fail to meet any of the criteria for remission. And the relapse criteria were defined according to the ACR 2006 clinical trial criteria (20). Disease relapse was characterized as a >25% increase in the Scr, or a 50% or more increase in proteinuria or active urine sediment characterized by >5 erythrocytes/HPF and/or cellular casts.

In the IgAN patients, CR was defined as a urine protein excretion (UPE) <0.3 g/d, along with normalization of all biochemical findings and a lack of worsening of renal function at the sixth month; PR was defined as at least a 50% reduction in UPE at the sixth month compared with baseline; NR was defined as a <50% reduction in UPE or an increase in UPE with or without renal deterioration after receiving 6 months of therapy; Relapse was defined as the reappearance of significant proteinuria, defined as >1.0 g/d and as a UPE increase of 50% from the lowest level of proteinuria after the start remission (21).

The estimated glomerular filtration rate (eGFR) was calculated with modified MDRD equation for Chinese patients:eGFR = 186 × (Scr/88.4)^−1.154^ × age^−0.203^ (×0.742, female) (22).

### Evaluation of Renal Biopsies

Renal biopsies of LN patients enrolled in this study were obtained and evaluated using the International Society of Nephrology/Renal Pathology Society (ISN/RPS) classification (23), and IgAN patients' renal biopsies were evaluated using the Oxford classification (24).

C4d staining was performed at Ren Ji Hospital by one of the investigators, who were blinded to the identity of the tissue. Biopsy samples were fixed in 4% paraformaldehyde (4% PFA) solution and embedded in paraffin. Paraffin sections (4 μm thick) were placed on positively charged slides and kept in a stove at 60°C for 1 h. Sections were deparaffinized and rehydrated through a series of xylene and graded alcohols. Antigen retrieval was performed by autoclave in a high-pressure sterilizer at 121°C for 5 min in 10 mM citrate buffer (pH 6.0). Endogenous peroxidase was blocked in 3% H_2_O_2_ for 10 min. The primary mouse anti-human C4d monoclonal antibody (LP69; Abcam) was applied at a dilution of 1:50 in primary antibody diluent (ZCIB01), and slides were incubated overnight at 4 °C. The slides were then incubated with goat anti-mouse IgG antibody (115-035-003; Jackson) for 1 h, and staining was visualized with the DAB (D8001; Sigma) for 5 min. Sections were washed with PBS (pH 7.4) between each step (3 times for 5 min each time). Finally, sections were counterstained with Mayer's hematoxylin, air-dried, cleared in xylene, and covered with glasses. Paraffin sections (4 μm thick) were also stained with periodic acid Schiff (PAS) and periodic acid-silver methenamine (PASM). Sections were imaged on a light microscope.

### Urine and Serum C4d Measurements

The serum and urine samples were collected soon after admission to the hospital. The collected samples were centrifuged at 3,000 g for 10 min to remove cellular debris. We aliquoted serum and urine supernatant into bar-coded cry-vials and stored the samples at −80°C until biomarker measurement. No additives or protease inhibitors were added.

We measured the serum and urine biomarker C4d with a commercially available ELISA kit (Dako, Denmark) following manufacturer's instruction. We measured urine creatinine by the modified Jaffe reaction. Personnel performing the biomarker measurements were blinded to each patient's clinical information. All biomarkers were measured from frozen aliquots that did not undergo any additional freeze-thaw cycles.

### Statistical Analysis

The statistical analysis was completed with SPSS, version 26.0. The normally distributed variables were expressed as the means ± SD and compared using a *t*-test or analysis of variance (ANOVA) as required. The non-parametric variables were expressed as the medians with 25^th^ and 75^th^ percentiles and compared using the Mann-Whitney U test. The Chi-square test was employed for the categorical variables. Cox proportional hazards regression was conducted with the candidate variables. The multivariable regression analysis was performed at the 0.1 level of significance for variables from a univariable analysis and used a stepwise forward selection procedure (25). Cumulative survival curves were derived using the Kaplan-Meier method, and the differences between survival curves were compared using the log-rank test.

## Results

### Baseline Clinical Characteristics and Histologic Findings

**Figure 1:**
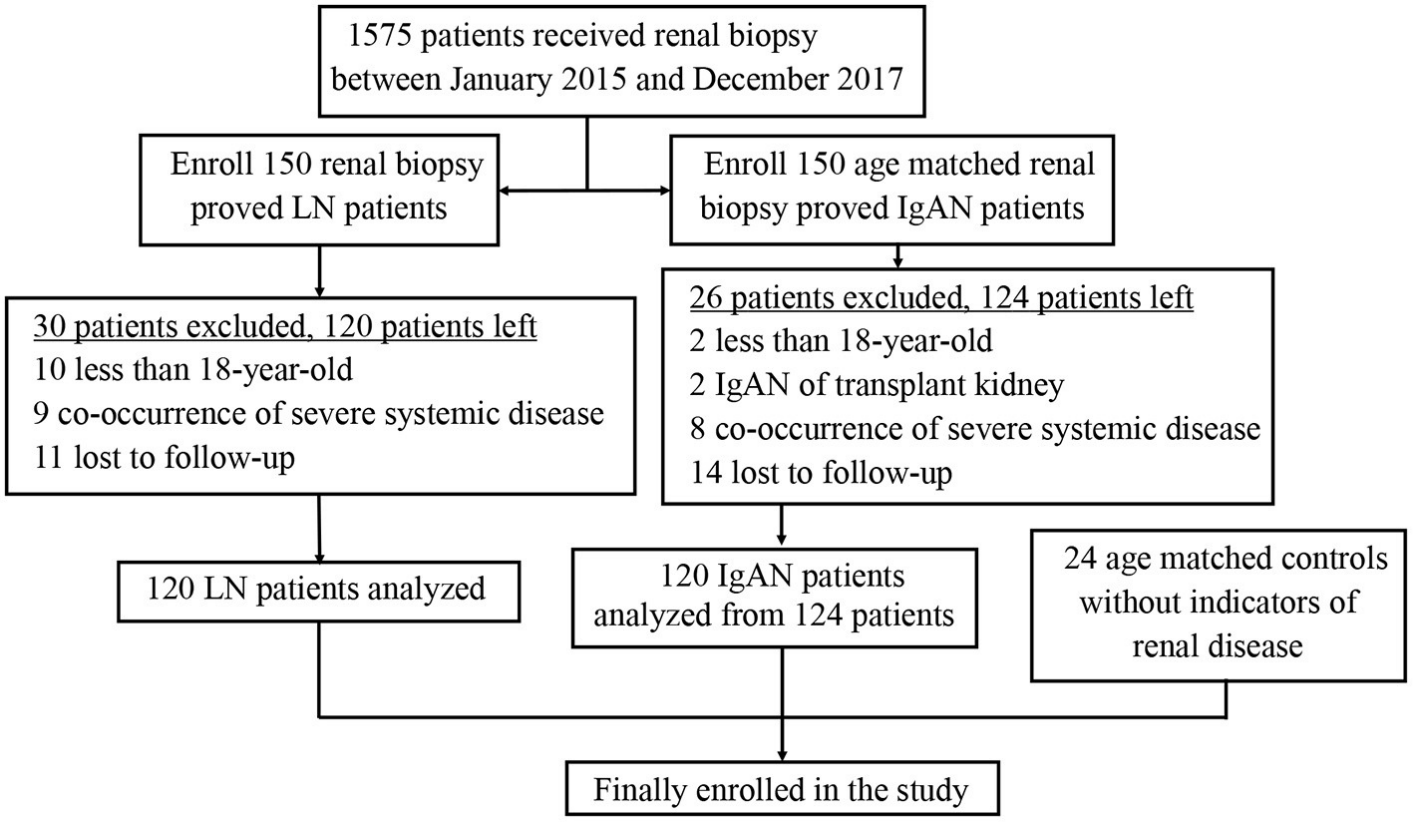
Flow diagram of the study design. A total of 120 LN patients, 120 IgAN patients and 24 healthy controls were enrolled into the study. LN, lupus nephritis; IgAN, IgA nephropathy.

A total of 264 subjects were finally included in the study cohort, including 120 LN patients, 120 IgAN patients and 24 healthy controls (Figure 1). Baseline characteristics of all participants were listed in Table 1. Subjects from all groups were matched in ages. There was no difference in follow-up duration between LN patients [38.5(30.3–60.8) months] and IgAN patients [45.0(30.5–57.0) months] (*P* > 0.05). Compared with IgAN patients, LN patients had lower serum C3 levels [1.10 (0.96–1.25) g/L vs. 0.37 (0.29–0.53) g/L, *P* < 0.05] and lower serum C4 levels [0.24(0.21–0.29) g/L vs. 0.08 (0.02–0.29) g/L, *P* < 0.05]. Besides, LN patients with C4d deposition in renal tissue were much more than IgAN patients (65.0% vs. 32.5%, *P* < 0.001). Patients with LN and IgAN showed comparable levels of serum C4d [23.60 (19.52–25.94) μg/ml vs. 23.43(19.37–25.93) μg/ml, *P* > 0.05] and urine C4d excretion [18.34 (15.59–25.05) μg/ml vs. 26.04(22.90–29.35) μg/ml, *P* > 0.05].

**Table 1:** Clinical and histologic characteristics at baseline for LN, IgAN and healthy control patients.

**Characteristics**	**Healthy control** **(***N*** = 24)**	**LN** **(*N* = 120)**	**IgAN (***N*** = 120)**
Age (years)	36.0 (23.5–50.4)	35.9 (20.3–55.5)	32.0 (21.6–45.1)
Gender: Female, *n* (%)	12 (50.0)	108 (90.0)	64 (53.3)
Follow-up (months)	—	38.5 (30.3–60.8)	45.0 (30.5–57.0)
Hb (g/L)	132.0(122.5–145.5)	101.0(86.5–115.5)	139.0 (128.5–151.5)
Scr (μmol/L)	60.2 (50.0–85.1)	62.0 (52.2–80.3)	88.0 (60.0–111.6)
eGFR (ml/min/1.73 m^2^)	105.9 (90.5–120.5)	105.6 (69.7–141.5)	85.8 (60.3–110.7)
Serum albumin (g/L)	39.5 (38.6–45.2)	33.6 (25.6–41.6)	38.5 (35.7–44.2)
anti-dsDNA antibody (IU/mL)	—	34.3 (10.1–69.3)	—
uRBC (/HP)	—	21.0 (15.6–100.2)	15.0 (8.1–143.5)
UPE (g/d)	—	1.38 (0.27–4.58)	1.05 (0.81–3.54)
Serum C3 level (g/L)	—	0.37 (0.29–0.53)	1.10 (0.96–1.25)
Serum C4 level (g/L)	—	0.08(0.02-0.29)	0.24(0.21–0.29)
Serum C4d level (μg/ml)	19.90 (15.07–23.33)	23.60 (19.52–25.94)	23.43 (19.37–25.93)
Urine C4d excretion (μg/ml)	16.23 (14.56–24.22)	18.34 (15.59–25.05)	26.04 (22.90–29.35)
Renal biopsy, classification		ISN/RPS pathologic classification, *n* (%)	Oxford classification, *n* (%)
	—	III, 20 (1.7)	M1, 70 (58.3)
	—	IV, 53 (44.2)	E1, 30 (25.0)
	—	III+V, 6 (5.0)	S1, 89 (74.2)
	—	IV+V, 19 (15.8)	T0/T1/T2, 68 (56.7)/35 (29.2)/17 (14.2)
	—	V, 22 (18.3)	C0/C1/C2, 62 (51.7)/53 (44.2)/5 (4.2)
**C4d deposition in renal tissue**			
C4d positive, n (%)	—	78 (65.0)	39 (32.5)

*The median (25^th^-75^th^ percentile) was shown if the variables did not show normal distributions, and n (%) was shown for the categorical variables. LN, lupus nephritis; IgAN, IgA nephropathy; Hb, hemoglobin; Scr, serum creatinine; eGFR, estimated glomerular filtration rate; uRBC, urine red blood cell; UPE, urine protein excretion*.

### Renal C4d Deposition Patterns in LN and IgAN Patients

**Figure 2:**
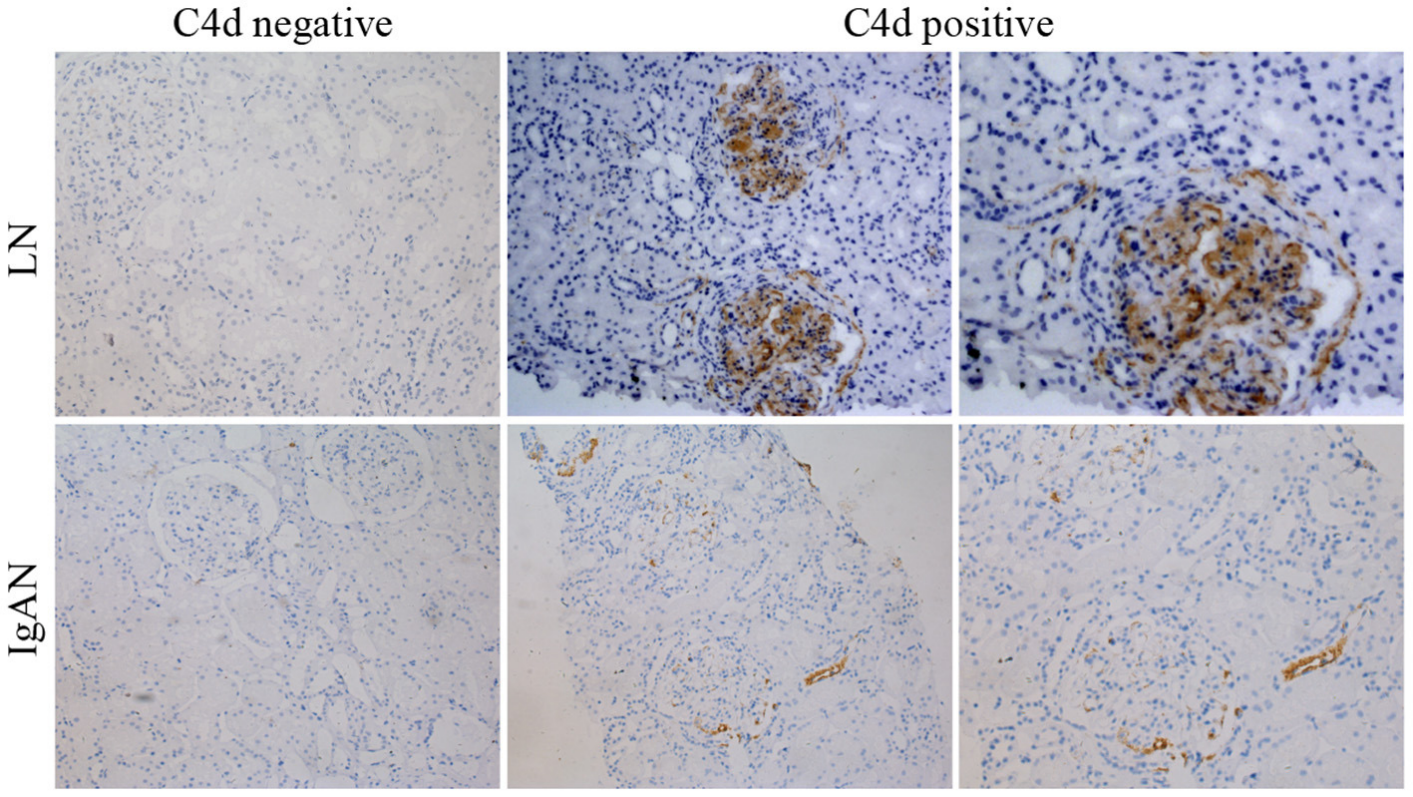
Representative immunohistochemical images showing negative or positive C4d deposition in renal tissue of LN and IgAN patients. The top panels were images for LN patients with negative (left panel) or positive (central and right panel) C4d deposition. The bottom panels were images for IgAN patients with negative (left panel) or positive (central and right panel) C4d deposition. ×200 (left and central panels). ×400 (right panels). LN, lupus nephritis; IgAN, IgA nephropathy.

C4d deposits could be found in glomerulus, tubules, interstitium and peritubular capillaries of renal tissue for both LN and IgAN patients. For LN patients, most of the C4d deposited in glomerulus (92.3%) and peritubular capillaries (19.2%) of renal tissue. For IgAN patients, most of C4d deposited in renal glomerulus (56.4%) and tubules (30.8%). Although both LN and IgAN patients had the most C4d distribution in renal glomerulus, the glomerular C4d deposition in LN patients was significantly more than IgAN patients (P <0.001). On the contrary, the LN patients had statistically less C4d deposition in renal tubules compared with IgAN patients (*P* = 0.003) (Table 2, Figure 2).

**Table 2:** The C4d distribution patterns in renal tissue for LN and IgAN patients.

**C4d distribution in renal tissue**	**LN (***n*** = 78)**	**IgAN** **(*n* = 39)**	***P*** **value**
Glomerulus, *n* (%)	72 (92.3)	22 (56.4)	<0.001
Tubules, *n* (%)	7 (9.0)	12 (30.8)	0.003
Arterioles, *n* (%)	4 (5.1)	3 (7.7)	0.890
Peritubular capillaries, *n* (%)	15 (19.2)	6 (15.4)	0.609

*P value: comparation between LN and IgAN group. LN, lupus nephritis; IgAN, IgA nephropathy*.

**Figure 3:**
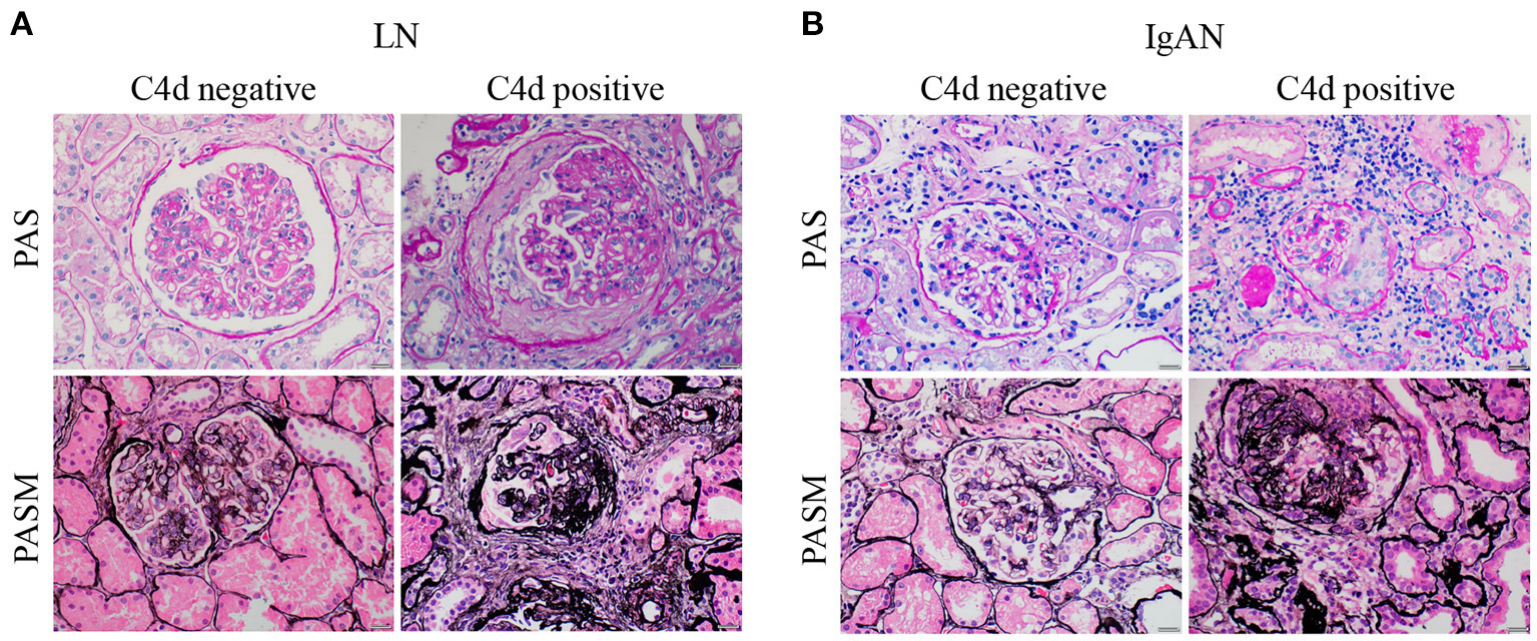
Representative periodic acid schiff (PAS) and periodic acid-silver methenamine (PASM) images of LN and IgAN patients with negative or positive renal C4d deposition. **(A)** The representative PAS (top panels) and PASM (bottom panels) images of LN patients with (right panels) or without (left panels) renal C4d deposition. **(B)** The representative PAS (top panels) and PASM (bottom panels) images of IgAN patients with (right panels) or without (left panels) renal C4d deposition. Scale bars: 20 μm. LN, lupus nephritis; IgAN, IgA nephropathy; PAS, Periodic Acid Schiff; PASM, periodic acid-silver methenamine.

C4d deposition were detected in various subtypes of LN but especially strong in class V membranous LN. Accordingly, IgAN patients with renal C4d deposition had a greater probability of tubulointerstitial fibrosis (T1/T2). While other Oxford score indices including mesangial hypercellularity (M1), endothelial hypercellularity (E1) lesions, segmental (S1) lesions and crescents (C1/C2) lesions had no significant correlations with renal C4d deposition (Table 3, Figure 3).

**Table 3:** Relationship between renal C4d deposition with the ISN/RPS pathologic classes for LN and the Oxford classification for IgAN.

**Pathologic classification**	**C4d positive**	**C4d negative**	***P*** **value**
**LN (ISN/RPS)**	***N*** **= 78**	***N*** **= 42**	
III, *n* (%)	10 (12.82)	10 (23.81)	0.123
IV, *n* (%)	30 (38.46)	23 (54.76)	0.086
III+V, *n* (%)	3 (3.85)	3 (7.14)	0.725
IV+V, *n* (%)	15 (19.23)	4 (9.52)	0.165
V, *n* (%)	20(25.64)	2(4.76)	0.005
**IgAN (Oxford-MESTC score)**	***N*** **= 39**	***N*** **= 81**	
Mesangial proliferation (M1), *n* (%)	24 (61.5)	46 (56.8)	0.621
Endocapillary proliferation (E1), *n* (%)	11 (28.2)	19 (23.5)	0.574
Segmental sclerosis (S1), *n* (%)	31 (79.5)	58 (71.6)	0.356
Interstitial fibrosis/Tubular atrophy (T1-T2), *n* (%)	22 (56.4)	30 (37.0)	0.045
Crescent (C1-C2), *n* (%)	20 (51.3)	38 (46.9)	0.654

*P value: comparation between C4d positive and C4d negative group. LN, lupus nephritis; IgAN, IgA nephropathy*.

### Correlation Between Renal C4d Deposition With Clinical Outcomes for LN and IgAN Patients

For disease treatment responses, it is indicated that LN patients with renal C4d deposition had less probability of sustained-remission (*P* = 0.002) and higher risk of relapse (*P* = 0.001) compared with C4d negative patients. While there was no significant relationship between renal C4d deposition with relapse for IgAN patients (*P* = 0.052). For sustained remission, C4d deposited IgAN patients were statistically less in comparison to C4d negative IgAN patients (*P* = 0.043) (Table 4). As far as the renal function outcome during the follow-up period, IgAN patients with renal C4d deposition were associated with more IgAN progression events compared with C4d negative ones (*P* < 0.001). For LN patients, the presence of renal C4d deposition was not statistically correlated with LN progression during the follow-up time (*P* = 0.812) (Table 5).

**Table 4:** Relationship between renal C4d deposition with responses to treatment of LN and IgAN patients.

**Responses to treatment**	**C4d positive**	**C4d negative**	***P*** **value**
**LN**	***N*** **= 78**	***N*** **= 42**	
Sustained-remission, *n* (%)	36 (46.15)	32 (76.19)	0.002
Non-response, *n* (%)	12 (15.38)	6 (14.29)	0.872
Relapse, *n* (%)	30 (38.46)	4 (9.52)	0.001
**IgAN**	***N*** **= 39**	***N*** **= 81**	
Sustained-remission, *n* (%)	24 (61.54)	64 (79.01)	0.043
Non-response, *n* (%)	6 (15.38)	9 (11.11)	0.713
Relapse, *n* (%)	9 (23.07)	8 (9.87)	0.052

*P value: comparation between C4d positive and C4d negative group. LN, lupus nephritis; IgAN, IgA nephropathy*.

**Table 5:** Relationship between renal C4d deposition with the disease progression of LN and IgAN patients during the follow-up period.

**Disease progression**	**C4d positive**	**C4d negative**	***P*** **value**
**LN**	***N*** **= 78**	***N*** **= 42**	
LN progression event, n(%)	28 (35.89)	16 (38.09)	0.812
**IgAN**	***N*** **= 39**	***N*** **= 81**	
IgAN progression event, n(%)	30 (76.92)	7 (8.64)	<0.001

*P value: comparation between C4d positive and C4d negative group. LN, lupus nephritis; IgAN, IgA nephropathy*.

### C4d as an Independent Predictor for Clinical Outcomes of LN and IgAN Patients

To further explore the correlation between renal C4d deposition with disease relapse of LN patients, renal C4d deposition and other variables were included into the univariate and multivariate Cox regression analysis and concluded that high baseline serum anti-dsDNA antibody (HR = 1.105, *P* = 0.034) and renal C4d deposition (HR = 1.007, *P* = 0.040) are independent predictors of disease relapse for LN patients (Table 6).

**Table 6:** Univariate and multivariate Cox regression analysis for predictors of the disease relapse in LN patients.

**Characteristics**	**Univariate analysis**			**Multivariate analysis**		
	**HR**	**95% CI**	***P* value**	**HR**	**95% CI**	***P* value**
Age (years)			0.280			
Gender (female vs. male)			0.360			
UPE (g/d)			0.095			
Scr (μmol/L)			0.361			
Serum albumin (g/L)			0.191			
anti-dsDNA antibody (IU/mL)	1.835	1.657–2.109	0.002	1.105	1.002–1.365	0.034
Urine C4d excretion (μg/ml)			0.125			
Serum C4d level (μg/ml)			0.741			
Renal C4d deposition	1.012	1.002–3.254	0.003	1.007	1.003–1.054	0.040

*Variables with P < 0.1 in the univariate Cox regression model were further analyzed in the multivariate Cox regression model. LN, lupus nephritis; UPE, urine protein excretion; Scr, serum creatinine*.

**Figure 4:**
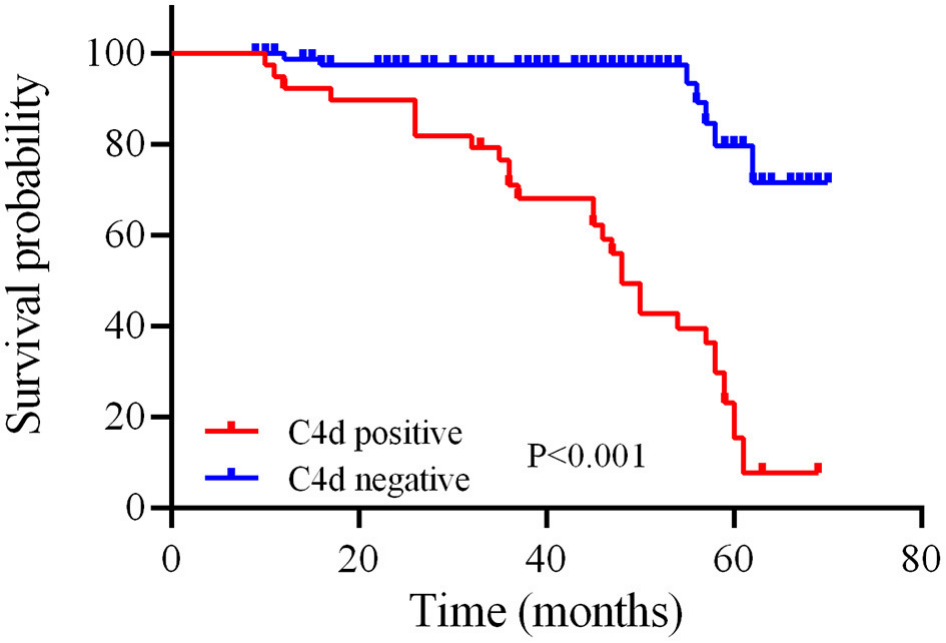
Relationship between C4d deposition in renal tissue and disease progression by time of IgAN patients. The red and blue line separately illustrate the survival probability over time for IgAN patients with positive or negative renal C4d deposition. Kaplan-Meier analysis with log-rank test revealed a significant difference between groups (*P* < 0.001).

As far as the long-term renal function outcome for IgAN patients, univariate and multivariate Cox regression analysis suggested that high baseline serum creatinine (HR = 1.010, *P* = 0.034) and renal C4d deposition (HR = 1.821, *P* = 0.040) are independent predictors of disease progression during the follow-up period for IgAN patients (Table 7). Similarly, Kaplan-Meier plot also indicated that renal C4d deposition is positive correlated with disease progression by time for IgAN patients (log-rank test, *P* < 0.001, Figure 4).

**Table 7:** Univariate and multivariate Cox regression analysis for predictors of disease progression during the follow-up period for IgAN patients.

**Characteristics**	**Univariate analysis**			**Multivariate analysis**		
	**HR**	**95% CI**	***P*** **value**	**HR**	**95% CI**	***P*** **value**
Age (years)			0.428			
Gender (female vs. male)	0.139	0.017–1.111	0.063			
UPE (g/d)	1.221	1.062–1.404	0.005			
Scr (μmol/L)	1.011	1.004–1.018	0.003	1.010	1.020–1.030	0.034
Serum albumin (g/L)	0.860	0.760–0.960	0.010			
Urine C4d excretion (μg/ml)	8.095	2.529–25.912	<0.001			
Serum C4d level (μg/ml)	1.661	1.152–2.393	0.007			
Renal C4d deposition	2.011	1.990–5.210	0.030	1.821	1.723–4.540	0.040

*Variables with P < 0.1 in the univariate Cox regression model were further analyzed in the multivariate Cox regression model. eGFR, estimated glomerular filtration rate; IgAN, IgA nephropathy; UPE, urine protein excretion; Scr, serum creatinine*.

## Discussion

The complement system plays significant roles in the pathogenesis of LN and IgAN (6, 12). As the complement-split product and a good marker of classical and lectin pathway activation, C4d is usually used to monitor humoral rejection in renal transplant biopsy samples (26). Several studies have confirmed that C4d is associated with the pathogenesis of both LN and IgAN (15–18). Nevertheless, studies directly comparing the screening and diagnostic value, and outcomes predicting function of renal C4d deposition between LN and IgAN are still relatively sparse. In the present study, we demonstrated that LN and IgAN patients had different C4d deposition proportions and patterns in renal tissue. Moreover, renal C4d deposition was especially strong in class V membranous LN and IgAN with tubulointerstitial fibrosis lesions. Furthermore, the renal C4d deposition was an independent predictor of disease relapse for LN patients and disease progression for IgAN patients respectively.

Among many immune related renal diseases, LN and IgAN are the two most common glomerular diseases (27). As the frequent complication of SLE, LN involves multiple pathogenic pathways including aberrant apoptosis, autoantibody production, immune complex deposition and complement activation. The classical ISN/RPS classification system of LN is mainly based on glomerular lesions. While recently studies indicated that tubulointerstitial lesions and vascular injury were also important indicators of LN and should be incorporated into the classification of LN (28). IgAN is defined by the presence of IgA-dominant or co-dominant immune deposits within glomeruli. The pathogenesis of IgAN is a multi-hit process, including the production of galactose-deficient IgA1, anti-glycan response, formation and deposition of IgA1-containing immune complexes. The immune complexes deposition in glomerular mesangium activates the complement pathway, stimulates mesangial cells, and induces secretion of cytokines, chemokines, and extracellular matrix proteins resulting in tubulointerstitial inflammation and fibrosis (29, 30).

As indicated above, LN and IgAN shared immune-complex related pathogenesis, while the specific mechanisms remain different. Nevertheless, coexistence of IgAN and SLE was found in some cases (8–10). Genetic studies have pointed out partial sharing of mechanisms of LN and IgAN. Genes like CFH, HLA-DRA, HLA-DRB1, PXK, BLK, UBE2L3 and MTMR3 are shared loci between IgAN and SLE/LN based on GWAS, which highlights pathways including MHC class-II antigen presentation, complement regulation, signaling by the BCR, autophagy, and ubiquitin/proteasome-dependent degradation shared between these two complex diseases (18, 27, 31). As a result, identification between the LN and IgAN for patients with kidney injuries remains significant. As the marker of complement activation, C4d is involved into the pathogenesis of both LN and IgAN. However, studies comparing the C4d between LN and IgAN remain sparse (15–18). In this situation, our study aims to directly compare C4d in LN and IgAN from several aspects and explore whether C4d could act as an important marker for differential diagnosis between IgAN and LN.

In our study, we observed that C4d was a shared renal deposition molecule and distributed differently between LN and IgAN patients. It is indicated that LN and IgAN had comparable levels of serum C4d and urine C4d excretion. However, LN patients with renal C4d deposition were much more than that of IgAN patients, which is consistent with previous studies (15–17). Moreover, other studies further indicated that plasma C4d levels correlated with renal C4d deposition in LN patients (32). Mesangial deposition of C4d was found to be an independent predictor of urinary C4d levels for IgAN patients (33). We also revealed that the renal C4d distribution patterns differed between LN and IgAN patients. Compared with IgAN patients, the LN patients were more likely to have C4d deposition in renal glomerulus and less likely in tubules. Some other studies even explored the specific location of C4d in glomeruli and extraglomerular parts. Previous studies indicated that C4d deposition was mainly in glomerular mesangium for IgAN patients (34–36). For LN patients, the renal C4d deposition was predominantly along the glomerular capillary loops and to a lesser extent in mesangium (15, 34, 37–39). Based on those findings, we could conclude that the differences and characters of renal C4d deposition pattern between LN and IgAN have made C4d a reliable screening and diagnostic tool to recognize and differentiate LN and IgAN.

In the present study, renal C4d deposition was especially strong in class V membranous LN and IgAN with tubulointerstitial fibrosis lesions. This result was similar with other studies. Kim et al. found a diffusely intense renal C4d deposition in class V membranous LN (15). For IgAN, tubulointerstitial fibrosis lesions were demonstrated to be the most consistent feature associated with renal C4d deposits in other studies (18, 40). Moreover, other studies also indicated that the renal C4d deposition significantly correlated with the presence of IgG, IgA, C3, C4, and C1q in LN patients (37, 38, 41). In IgAN patients, glomerular C4d staining was found to be associated with mesangial IgM deposition (35). Based on those findings, it is suggested that C4d could also act as a factor to facilitate the recognition of worse pathologic features for LN and IgAN patients, which is beneficial for the therapeutic regimens to be adopted, thus promoting the clinical prognosis of the patients.

It is demonstrated that LN patients with renal C4d deposition had a higher risk of relapse after treatment compared with the C4d negative patients. While there was no significant relationship between renal C4d deposition and LN progression. Ding et al. concluded that arteriolar C4d deposition was associated with worse renal outcomes, while C4d deposition in other renal compartments did not influence patient renal outcomes (39), which is instructive for our study. However, restricted by the fact that the cases in each subgroup of LN patients are too less to conduct the analysis, we cannot further confirm this conclusion. Moreover, it could also be attributed to the follow-up period, which was not long enough for LN patients to experience renal function deterioration. As previous studies have proved the definitive role of relapse with LN progression (42). Thus we could speculate that the relationship between renal C4d deposition and LN progression might be found when extending the follow-up duration.

In the present study, we demonstrated that renal C4d deposition is an independent predictor for IgAN progression during the follow-up period, which is similar with most of other studies indicated that C4d deposition was associated with lower eGFR, worse proteinuria, and worse renal survival for IgAN patients (17, 18, 43–45). Previous studies indicated that renal survival at 5 and 20 years was 76 and 28% respectively for IgAN patients. In consideration of the follow-up period in our study, we finally selected a decline of eGFR by more than 20% or end-stage kidney disease as the indicators for the disease progression event. There was no significant correlation between renal C4d deposition and relapse after treatment for IgAN patients, which is different from LN patients.

There were also some limitations to this study. Firstly, the study population was under-represented in the general population. This is a single-center study. Bias in patient selection might therefore have been unavoidable. Secondly, restricted by the indications of renal biopsy, we had no access to renal tissues from healthy control, which make it difficult for us to explore C4d deposition in healthy kidneys. Moreover, the relationship between C4d stratified by the deposition sites in kidney, severity and kinds of pathologic lesions with clinical outcomes were not explored in this study as the patients with the onset of outcome events in each subgroup were too less to conduct the analysis. Finally, as stated above, the longer follow-up period might contribute more valuable information.

## Conclusions

In summary, we demonstrated that renal C4d deposition was shared between LN and IgAN patients, while the depositing proportion and patterns differed significantly between LN and IgAN. The renal C4d deposition was especially strong in class V membranous LN and IgAN with tubulointerstitial fibrosis lesions, meanwhile, acted as an independent predictor of relapse for LN patients and disease progression for IgAN patients.

## Data Availability Statement

The datasets presented in this article are not readily available as further analysis is going to be done based on it. Requests to access the datasets should be directed to Shan Mou, moushanrenji@126.com.

## Ethics Statement

The studies involving human participants were reviewed and approved by Ethics Committee of Renji Hospital affiliated to Shanghai Jiao Tong University School of Medicine. The patients/participants provided their written informed consent to participate in this study.

## Author Contributions

SM, QW, and CQ: designed research. XY, YY, XS, HP, XC, LC, MZ, YX, ZN, CQ, QW, and SM: performed research. All authors contributed to the article and approved the submitted version.

## Funding

This study was supported by Ministry of Science and Technology of China (2017YFE0110500), National Natural Science Foundation of China (81770668, 81970574). The study was also sponsored by Shanghai Municipal Health Commission (ZXYXZ-201904), Shanghai Jiao Tong University School of Medicine (18ZXY001) as well as Shanghai Outstanding Academic Leaders Plan.

## Conflict of Interest

The authors declare that the research was conducted in the absence of any commercial or financial relationships that could be construed as a potential conflict of interest.

## Publisher's Note

All claims expressed in this article are solely those of the authors and do not necessarily represent those of their affiliated organizations, or those of the publisher, the editors and the reviewers. Any product that may be evaluated in this article, or claim that may be made by its manufacturer, is not guaranteed or endorsed by the publisher.
